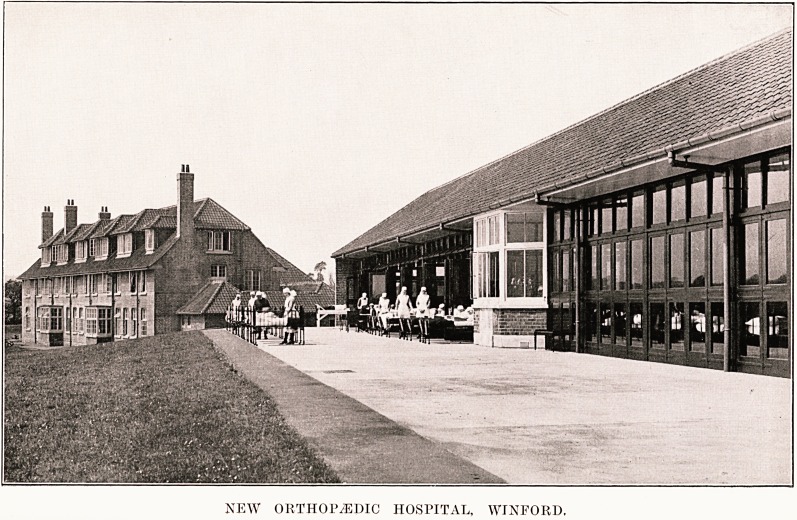# Editorial Notes

**Published:** 1930

**Authors:** 


### Editorial Notes

**Death of Dr. Bridges.**

Dr. Bridges's death has robbed English letters of a singular personality. His appointment as Poet Laureate in 1913 came as a surprise. His poetry was only known to a few, and his other contributions to literature were still less widely known. Now at the end of a long life his energies, his inspirations and the music of his tongue are more fully acknowledged. He was a Poet Laureate worthy to rank with the few holders of that office who have deserved the laurel crown.

Our English speech will be the richer as well as the purer for Bridges's efforts. His education was unquestionably orthodox and stereotyped ; Eton and Oxford might be expected to produce a versifier who would cling to the classical models—and Robert Bridges knew the classical models and used them well. Judged from current journalism Eton and Oxford might only seem likely to furnish drawling affectation in their poets. But somehow through all of Bridges poetry there runs a music that is the rarest of all the qualities in English Poetry.

England has had readable poets enough and to spare, but the singable ones can scarcely be found. Robert Bridges has a marvellous sense of the music in words, but then he was a musician as well as a poet. This rare combination made him able to fashion such a lyric as " The Stranger " (Whither oh splendid ship thy white sails spreading). Not even his medical education can take credit for imparting this quality to his verses. Yet he may have owed something to an earlier doctor-poet. The link between him and Thomas Campion is unmistakeable. Of all doctors turned poets none else excelled in both callings.

Keats, Goldsmith and Cowley, high as they stand as poets, were of no medical account; Schiller practised for a time as an Army Surgeon, and abandoned medicine for letters as soon as he was able. Of the rest Garth, Akenside and Co.—they barely rank with the minimal poets.

Bridges practised medicine long enough to prove his worth; he was a Radcliffe travelling fellow, then Casualty Physician at St. Bartholomew's, Assistant Physician at Great Ormond Street, and Physician at the Great Northern Hospital. When forty years old he retired from medicine as he had long planned to do, and his second life in letters began.

Campion, the Elizabethan, who in his *Two books of Airs* tells the reader that he chiefly aimed to couple his words and notes lovingly together, practised as a physician, and when in 1615, Sir Thomas Monson was confined to the Tower in connection with the murder of Sir Thomas Overbury, Campion acted as his medical attendant there, so that he was actively engaged in medicine until within five years of his death.

The resemblance between Campion and Bridges extends further than to their medical training and poetic tastes. Like Bridges, Campion had explored the art of prosody and published in 1602 his observations on the art of English poesy; either Campion or Bridges might have written of our English

NEW ORTHOPÆDIC HOSPITAL, WINFORD.

syllables, "the sound of them in a verse is to be valued and not their letters." Surely Bridges echoed some of the music of Campion's line, "Could I enchant, and that it lawful were," when he wrote in his "Elegy," "Could I forget then were the fight not hard."

If English medicine should claim two representative poets who were likewise doctors, the names of Campion and Bridges must stand alone.

**The New Hospital at Winford.**

ON May 31st, the new Orthopædic Hospital at Winford, formed by amalgamation of the Bristol Orthopædic Hospital with the Bristol Crippled Children's Society, was opened by Prince George. Fortunately the rain held off until the ceremony was over, and the large company of guests had an admirable opportunity of seeing for themselves the kind of hospital that is being developed on the new site. (It will be remembered that less than two years ago the foundation stone was laid by the Duke and Duchess of York.) The nature of the work that is contemplated was explained by Miss F. M. Townsend, J.P., Chairman of the Hospital, and also by its President, the Lord Mayor of Bristol. The buildings were described by the architect, Sir George Oatley. These consist of one ward block holding fifty-six beds, to which it is hoped to add at least one other block as soon as funds permit; a treatment block which includes an operating theatre, also plaster, light, X-ray, and exercise rooms; an administrative block housing the nursing and domestic staff and the resident medical officer; an engine house for generating the power which heats and lights the hospital; and a laundry. The hospital has its own water supply. The site was chosen with care as the best available within a radius of six miles from the centre of Bristol, and sixty-seven acres were purchased so as to afford room for extensions to the city hospitals, if and when these are desired. There is still a debt of about £18,000 on these buildings, which it is hoped will be met within the remainder of the current year.

The Medical and Surgical Staff of the Hospital is as follows:—

Honorary Consulting Staff.—*Physicians:* F. J. Poynton, M.D., F.R.C.P., Carey F. Coombs, M.D., F.R.C.P., R. C. Clarke, M.B., M.R.C.P. *Surgeons:* Sir Robert Jones, Bart., K.B.E., C.B., F.R.C.S., E. W. Hey Groves, M.S., F.R.C.S., G. R. Girdlestone, B.Ch., F.R.C.S.

Honorary Staff.—*Physicians:* C. E. K. Herapath, M.D., C. B. Perry, M.D., M.R.C.P. *Surgeon:* H. Chitty, M.S., F.R.C.S., *Dental Surgeon:* Clifford Wing, L.D.S., R.C.S. *Anæsthetist:* A. L. Flemming, M.R.C.S., L.R.C.P. *Radiologist:* T. B. Wansbrough, M.B., Ch.B., D.M.R.E. *Pathologist:* A. L. Taylor, M.D. *Resident Medical Officer:* E. M. Redman, M.B., Ch.B.

**The Liverpool Medico-Chirurgical Journal.**

FOR some years—to be precise, from the end of 1916 to the middle of last year—the publication of the *Liverpool Medico-Chirurgical Journal* was suspended. That it has been revived is all to the good. It was always one of the best of the British Journals, and the members that mark its renewed activity are fully up to the high standard that we learnt to expect of it. The first article is one on William Harvey, appropriately written by Professor John Hay, whose services to his own medical school are no less conspicuous than those which he has rendered to the advancement of cardiology. This is followed by a number of interesting articles, and the second issue—the journal appears twice a year—is equally good. We congratulate the Editors, Drs. R. W. MacKenna and Robert Coope, on their achievement, and wish them continued success.

## Figures and Tables

**Figure f1:**